# Integrating multimedia training and microteaching in school-based oral health promotion: a mixed-methods study among primary school teachers and students in Gorontalo, Indonesia

**DOI:** 10.3389/froh.2026.1728222

**Published:** 2026-02-06

**Authors:** Selviawaty Sarifuddin Panna, Ayub Irmadani Anwar, Irfan Sugianto, Ichlas Nanang Afandi, Marhamah Firman Singgih, Nurlinda Hamrun

**Affiliations:** 1 Faculty of Dentistry, Hasanuddin University, Makassar, Indonesia; 2 Department of Dental Public Health, Faculty of Dentistry, Hasanuddin University, Makassar, Indonesia; 3 Departement of Oral and Maxillofacial Radiology, Faculty of Dentistry, Hasanuddin University, Makassar, Indonesia; 4 Department of Psychology, Faculty of Medicine, Hasanuddin University, Makassar, Indonesia; 5 Department of Pediatric Dentistry, Faculty of Dentistry, Hasanuddin University, Makassar, Indonesia; 6 Department of Oral Biology, Faculty of Dentistry, Hasanuddin University, Makassar, Indonesia

**Keywords:** Indonesia, microteaching, mixed methods, multimedia training, Oral Health, school-based intervention

## Abstract

**Background:**

Oral health is an essential component of overall well-being. Despite ongoing public health education efforts, childhood dental caries remain highly prevalent in Indonesia, particularly in resource-limited school settings. Teachers play a strategic role as change agents in shaping children's oral health knowledge, attitudes, and behaviors; however, their capacity to deliver effective oral health education is often underdeveloped. This study evaluated the impact of multimedia-assisted training and microteaching on teachers' facilitation, and its subsequent effect on students' oral health status and oral health–related knowledge, attitudes, and behaviors.

**Methods:**

A mixed-methods sequential explanatory design was employed involving 582 primary school students and 16 teachers from public elementary schools in Pohuwato District, Gorontalo Province, Indonesia. Teachers were allocated into two intervention groups: (1) multimedia training combined with microteaching (*n* = 8) and (2) multimedia training only (*n* = 8). Students' oral health outcomes were assessed over a six-month period using the Decayed, Missing, and Filled Teeth (DMFT) index and the Oral Hygiene Index Simplified (OHI-S). Teachers' knowledge, attitudes, and practices were evaluated using validated questionnaires. Qualitative data were collected through focus group discussions with teachers in the multimedia + microteaching group.

**Results:**

Both intervention groups demonstrated significant improvements in students' OHI-S and DMFT scores (*p* < 0.05), with the multimedia + microteaching group showing superior outcomes. Teachers’ knowledge and instructional performance improved significantly following the intervention (*p* < 0.01). Qualitative findings indicated that microteaching enhanced teachers' confidence, consistency, and creativity in delivering oral health education, contributing to more sustained behavioral change among students.

**Conclusion:**

Integrating multimedia training with microteaching strengthens teachers' pedagogical competence and leads to measurable improvements in students' oral hygiene and oral health–related behaviors. This school-based approach offers a replicable and scalable model for oral health promotion in low-resource educational settings.

## Introduction

Oral health is a fundamental determinant of quality of life and overall health, particularly among school-aged children (1, 2). In Indonesia, the prevalence of dental caries remains high despite various public health campaigns, reflecting persistent challenges related to socioeconomic disparities, inadequate oral hygiene practices, and limited access to preventive dental services (2, 3). These challenges are especially pronounced in resource-limited school environments, where structured oral health education is often insufficient (4).

In Indonesia, the burden of dental caries among school-aged children remains alarmingly high. National data from the Indonesian Basic Health Research (Riskesdas) reported that approximately 57.6% of children aged 5–9 years and 51.9% of children aged 10–14 years experienced dental caries, with untreated caries being the most prevalent oral condition (2). Regional studies from eastern Indonesia have similarly reported high caries prevalence, highlighting persistent disparities in access to preventive oral health services, particularly in rural and resource-limited settings (3, 5).

Schools represent a strategic setting for oral health promotion, as children spend a significant portion of their time in educational environments (2). Teachers, as daily influencers and role models, are well positioned to act as oral health promoters and to reinforce positive health behaviors among students (6). Previous studies have demonstrated that teacher-led health education interventions can improve students' oral health knowledge and behaviors; however, many teachers lack adequate training, confidence, and pedagogical skills to deliver effective oral health education consistently (5, 7).

Multimedia-based learning has emerged as an effective educational approach in health promotion, offering interactive visual and auditory content that enhances learner engagement and knowledge retention (8, 9). Several studies have shown that multimedia-assisted education can improve oral health knowledge and hygiene practices among children (10, 11). Nevertheless, multimedia tools alone may be insufficient if teachers are not adequately prepared to facilitate learning and translate knowledge into classroom practice (7).

Microteaching is a pedagogical strategy designed to strengthen teaching skills through structured practice, feedback, and reflection. By allowing teachers to rehearse instructional delivery in a controlled environment, microteaching has been shown to enhance teaching confidence, communication skills, and instructional consistency (7, 12). Despite its documented benefits in educational and health professional training, microteaching has been rarely integrated into school-based oral health promotion programs, particularly in low-resource settings (6, 12).

To address these gaps, the present study integrates multimedia-assisted training with microteaching to strengthen teachers' facilitation capacity in delivering oral health education. Using a mixed-methods approach, this study aimed to evaluate the combined effect of these interventions on teachers' instructional competence and students' oral health outcomes, including clinical indicators and oral health–related knowledge, attitudes, and behaviors. By situating the intervention within a real-world, resourcelimited school context, this study seeks to contribute evidence for scalable and sustainable oral health promotion strategies in primary schools.

## Materials and methods

### Study design

This study employed a mixed-methods sequential explanatory design, integrating quantitative and qualitative approaches to comprehensively evaluate the impact of teacher-based oral health promotion interventions. The quantitative component assessed changes in students’ oral health outcomes and teachers' knowledge, attitudes, and practices before and after the intervention. The qualitative component was conducted subsequently to explore teachers' experiences and perceptions regarding the implementation of multimedia training combined with microteaching, thereby providing contextual explanations for the quantitative findings.

Given the limited number of participating teachers (*n* = 16), teacher-level quantitative outcomes were interpreted as exploratory rather than confirmatory.

### Study setting and participants

The study was conducted in public primary schools in Pohuwato District, Gorontalo Province, Indonesia, from July 2024 to January 2025. This district represents a resource-limited educational setting with limited access to routine school-based oral health promotion programs.

### Study participants

Participants consisted of 16 primary school teachers and 582 students enrolled in grades 3–4 from selected public primar schools.

### Sampling and recruitment

A purposive sampling strategy was employed to select schools based on accessibility, willingness to participate, and approval from school authorities. Teachers were recruited from participating schools and allocated to intervention groups at the school level to minimize contamination between groups. All eligible students from participating classes were invited to take part in the study following parental consent.

### Inclusion and exclusion criteria

Teachers' inclusion criteria included teachers who were actively teaching grades 3–4 at the selected schools, willing to participate in the training and evaluation sessions, and who provided written informed consent.

Teachers were excluded if they were absent during the training sessions or did not complete the intervention.

Students' inclusion criteria included students enrolled in grades 3–4 who were physically and cognitively able to participate in oral examinations and whose parents or legal guardians provided written informed consent. Students were excluded if they had systemic conditions that could affect oral health outcomes or were absent during baseline or follow-up data collection.

A detailed overview of the intervention components and implementation procedures is presented in Table 1.

**Table 1:** Intervention procedures.

Component	Multimedia training + microteaching	Multimedia training only
Target participants	Primary school teachers (*n* = 8)	Primary school teachers (*n* = 8)
Training approach	Multimedia-based education combined with microteaching	Multimedia-based education
Training content	Oral anatomy; dental caries etiology; toothbrushing techniques; diet and oral health; preventive oral health behaviors	Same content as intervention group
Educational materials	Animated videos, instructional slides, visual demonstrations	Animated videos, instructional slides, visual demonstrations
Training duration	2 sessions (60–90 min per session)	2 sessions (60–90 min per session)
Microteaching sessions	Yes – structured teaching simulations with peer feedback and facilitator reflection	Not applicable
Microteaching duration	1–2 cycles per teacher (20–30 min per cycle)	Not applicable
Facilitators	Dental public health professionals and academic staff	Dental public health professionals and academic staff
Implementation setting	Public primary schools, Pohuwato District	Public primary schools, Pohuwato District
Follow-up period	6 months	6 months
Outcome measures	Students’ DMFT and OHI-S; teachers’ knowledge, attitudes, and practices; qualitative insights	Students’ DMFT and OHI-S; teachers’ knowledge, attitudes, and practices

The intervention components for each study arm are summarized in Table 1 to enhance clarity and comparability

Teachers were allocated into two intervention groups:

### Intervention 1: multimedia training + microteaching (*n* = 8 teachers)

Teachers in this group received multimedia-based oral health training followed by structured microteaching sessions. The multimedia training covered basic oral anatomy, dental caries etiology, toothbrushing techniques, dietary factors related to oral health, and preventive oral health behaviors for school-aged children. Educational materials included animated videos, instructional slides, and visual demonstrations.

The microteaching component consisted of structured teaching simulations in which teachers practiced delivering oral health education to peers, followed by facilitated feedback and reflective discussion. Each microteaching cycle lasted approximately 20–30 min, and teachers participated in one to two cycles to refine instructional strategies and classroom delivery.

### Intervention 2: multimedia training only (*n* = 8 teachers)

Teachers in this group received multimedia-based oral health training using the same content, materials, and duration as Intervention 1 but did not participate in microteaching sessions.

All training sessions were facilitated by dental public health professionals and academic staff from Universitas Hasanuddin. Training delivery was standardized to ensure consistency across intervention groups.

### Facilitators

All training sessions were facilitated by dental public health professionals and academic staff from Universitas Hasanuddin with expertise in oral health promotion and health education. Facilitators standardized the training delivery to ensure consistency across intervention groups.

### Outcome measures

Clinical oral examinations were conducted at two predefined time points: baseline (Month 0) and endline after six months of intervention (Month 6).

### Students' oral health outcomes

Students' oral health status was assessed using the Decayed, Missing, and Filled Teeth (DMFT) index and the Oral Hygiene Index Simplified (OHI-S). Clinical examinations were conducted at baseline and six months post-intervention by trained examiners using standardized World Health Organization criteria.

### Teachers' knowledge, attitudes, and practices

Teachers' knowledge, attitudes, and practices related to oral health education were assessed using validated questionnaires adapted from previously published instruments assessing teacher-based oral health promotion and health education practices (5, 6). The questionnaires were administered before and after the intervention.

### Qualitative data collection

Qualitative data were collected through a focus group discussion (FGD) with teachers from the multimedia training plus microteaching group following completion of the intervention. The discussion explored teachers' experiences, perceived benefits, challenges, and feasibility of implementing oral health education in the school setting. The FGD was audio-recorded, transcribed verbatim, and anonymized prior to analysis.

Coding was conducted manually without the use of qualitative analysis software.

### Data analysis

#### Quantitative analysis

Prior to analysis, data distribution was assessed using the Shapiro–Wilk test. As the data were not normally distributed, non-parametric statistical tests were applied. Within-group changes over time were analyzed using the Friedman test, while pre–post comparisons involving two time points were assessed using the Wilcoxon signed-rank test. All analyses were conducted using IBM SPSS Statistics version 25, with statistical significance set at *p* < 0.05.

#### Qualitative analysis

Qualitative data were analyzed using thematic analysis. Transcripts were independently coded by two researchers to identify initial codes and emerging themes. Discrepancies were resolved through discussion until consensus was achieved. Final themes were developed inductively based on recurring patterns in the data.

### Ethical considerations

Ethical approval was obtained from the Health Research Ethics Committee, Universitas Hasanuddin (Approval No. 0069/PL.09/KEPK FKG-RSGM UNHAS/2024). Written informed consent was obtained from all teachers and from parents or legal guardians of participating students. The study was conducted in accordance with the Declaration of Helsinki and local ethical regulations.

## Results

**Figure 1:**
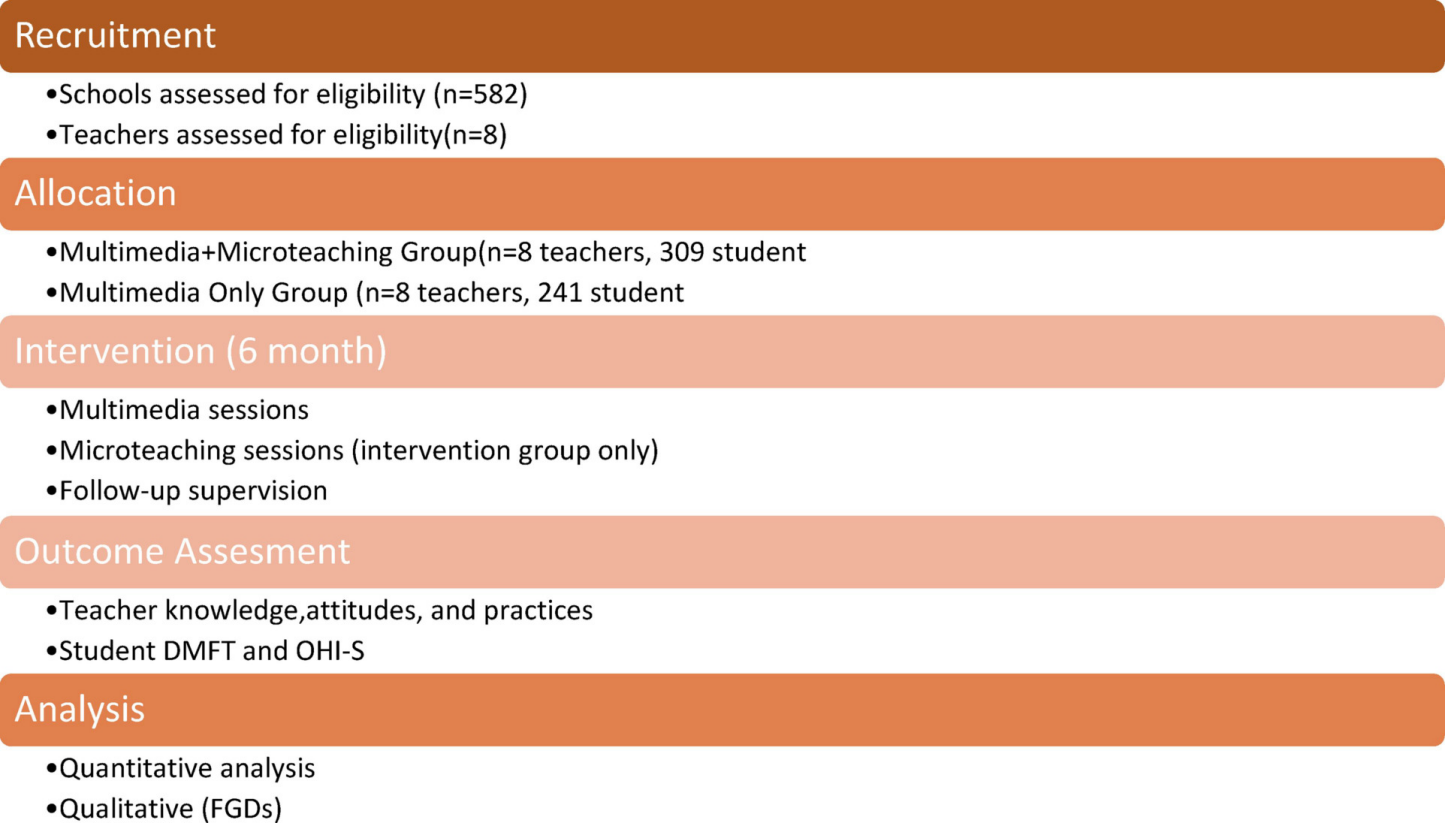
Study flow diagram. Study flow illustrating participant recruitment, school-level group allocation, intervention delivery, and outcome assessment over the six-month study period.

**Figure 2:**
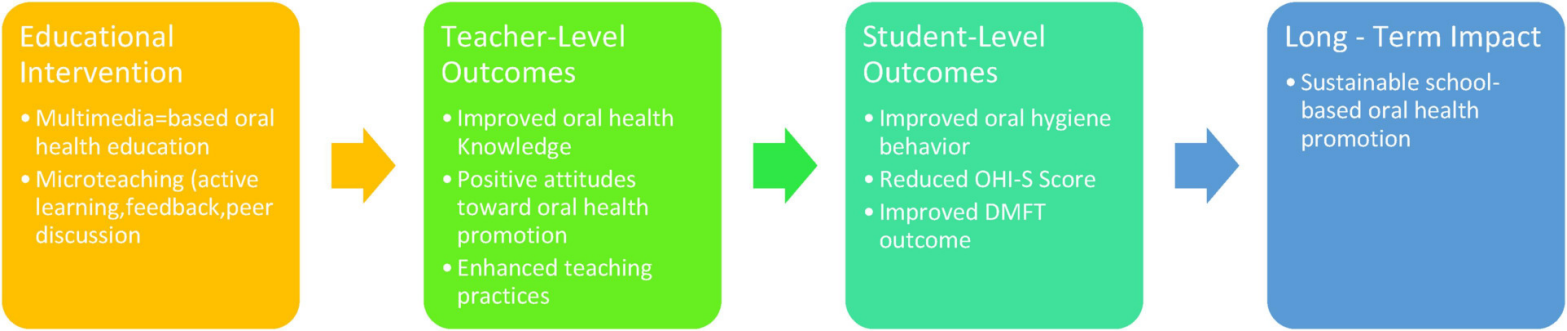
Conceptual Framework of the Intervention. Conceptual framework illustrating the hypothesized pathways through which multimedia-based education with microteaching enhances teachers’ knowledge, attitudes, and practices, leading to improved oral health outcomes among students.

**Figure 3:**
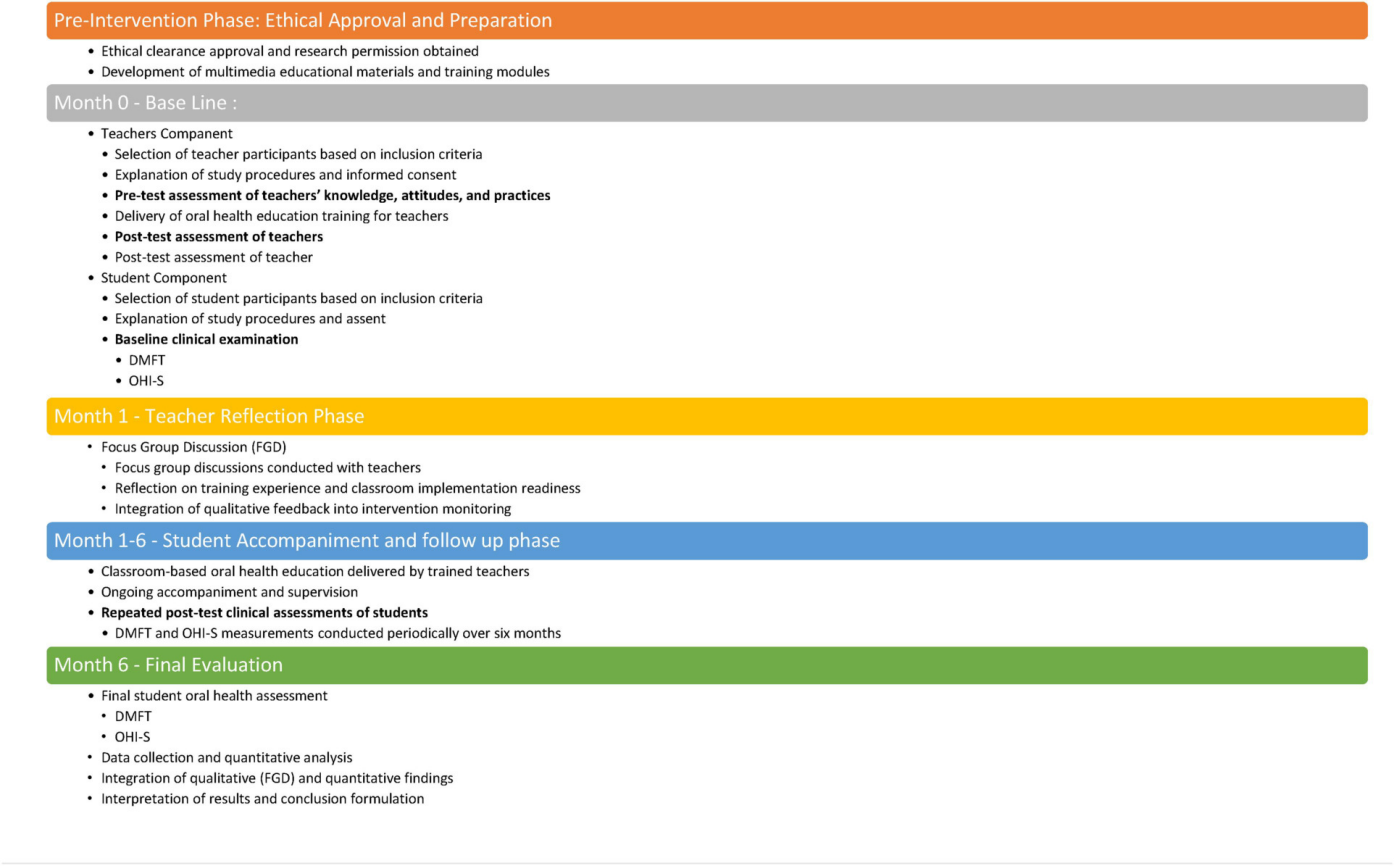
Intervention Timeline (6 months). Intervention timeline illustrating ethical preparation, multimedia development, teacher training and evaluation, focus group discussions, and six-month student accompaniment with repeated oral health assessments.

The study flow, conceptual framework, and intervention timeline are illustrated in Figures 1–3.

### Participant characteristics

A total of 582 primary school students and 16 teachers participated in the study. All participants completed both baseline and six-month post-intervention assessments. No participant attrition or missing data were observed during the follow-up period, and therefore all participants were included in the final analysis.

Teachers were equally distributed between the two intervention groups: multimedia training combined with microteaching (*n* = 8) and multimedia training only (*n* = 8). Students in both groups were comparable at baseline with respect to oral health indicators.

The primary analysis focused on within-group changes over time, as the study was designed to evaluate pre–post improvements following each intervention. Between-group comparisons were therefore interpreted descriptively.

### Changes in students' oral health outcomes

#### Oral hygiene Status (OHI-S)

Both intervention groups demonstrated significant improvements in students' oral hygiene status over the six-month follow-up period. In the multimedia training plus microteaching group, the mean OHI-S score decreased from 3.6 ± 0.8 at baseline to 1.9 ± 0.4 post-intervention (*p* < 0.001). The multimedia-only group also showed a significant reduction in OHI-S scores (*p* < 0.05), although the magnitude of improvement was greater in the combined intervention group (Table 2).

**Table 2:** Changes in students’ oral health and teachers’ knowledge and behavior scores.

Parameter	Baseline	Post-intervention	*p*-value
OHI-S (Multimedia + Microteaching)	3.6 ± 0.8	1.9 ± 0.4	<0.001
DMFT (Multimedia + Microteaching)	2.3 ± 0.5	1.1 ± 0.4	<0.001
Teachers’ knowledge score	65.2 ± 10.3	89.5 ± 8.1	<0.01
Teachers’ behavior score	62.8 ± 9.5	86.3 ± 7.7	<0.01

#### Dental caries experience (DMFT)

A significant reduction in DMFT scores was observed in both intervention groups. Students in the multimedia training plus microteaching group experienced a decrease from 2.3 ± 0.5 to 1.1 ± 0.4 (*p* < 0.001). Although students in the multimedia-only group also showed statistically significant improvements, post-intervention DMFT scores remained higher than those observed in the combined intervention group (Table 2).

### Changes in teachers' knowledge, attitudes, and practices

Teachers' knowledge and instructional practices related to oral health promotion improved significantly following the intervention. Mean knowledge scores increased from 65.2 ± 10.3 at baseline to 89.5 ± 8.1 post-intervention (*p* < 0.01). Similarly, instructional behavior scores increased from 62.8 ± 9.5 to 86.3 ± 7.7 (*p* < 0.01), with improvements being more pronounced among teachers who participated in the microteaching sessions (Table 2).

### Qualitative findings

Thematic analysis of focus group discussions identified three main themes:

(1) Microteaching as a key mechanism for translating knowledge into classroom practice,
(2) Transformation of teachers' roles into oral health facilitators, and
(3) Practical scalability and sustainability in resource-limited school settings.

Qualitative findings further supported the quantitative results by demonstrating that microteaching enhanced teachers’ confidence, consistency, and instructional effectiveness, which contributed to more sustained oral health–related behavior change among students.

1. Microteaching as a Key Mechanism for Translating Knowledge into Classroom Practice.

Representative quotes:

“Watching educational videos alone was helpful, but practicing how to teach oral health through microteaching was what truly changed how I explained the material in class.” (Teacher 1)

“Practicing in front of colleagues helped me anticipate students’ questions and respond more confidently during actual teaching.” (Teacher 3)

(1) Transformation of Teachers' Roles into Oral Health Facilitators

Representative quotes:

“After the training, I no longer felt like I was just delivering information. I felt responsible for guiding students’ daily oral health habits at school.” (Teacher 4)

“Because teachers are present every day, we were able to consistently remind students about proper toothbrushing, which made the behavior easier to maintain.” (Teacher 5)

(1) Practical Scalability and Sustainability in Resource-Limited School Settings

Representative quotes:

“This approach is realistic for schools like ours because it does not require frequent visits from dentists, yet teachers can continue the program independently.” (Teacher 2)

“Oral health education became easier to integrate into daily school routines after we learned how to deliver it effectively through microteaching.” (Teacher 6)

Qualitative findings from focus group discussions further supported the quantitative results. Three main themes emerged, highlighting the role of microteaching in translating knowledge into classroom practice, transforming teachers' roles as oral health facilitators, and enabling scalable and sustainable implementation in resource-limited school settings.

Between-group comparisons, effect sizes, and confidence intervals were interpreted descriptively due to the quasi-experimental design and exploratory nature of the analyses.

## Discussion

This mixed-methods study demonstrated that integrating multimedia-based training with microteaching produced superior improvements in both students' oral health outcomes and teachers' instructional competence compared with multimedia training alone. The combined intervention resulted in greater reductions in students' OHI-S and DMFT scores, alongside significant improvements in teachers' knowledge, attitudes, and instructional practices. These findings highlight the added pedagogical value of microteaching in strengthening school-based oral health promotion programs.

### Students' oral health outcomes

The significant improvements observed in students' oral hygiene status and dental caries experience align with previous evidence indicating that school-based oral health interventions can positively influence children's oral health behaviors and clinical outcomes. Studies conducted in various settings have shown that teacher-led oral health education contributes to improved toothbrushing practices and reduced plaque accumulation among school-aged children (3, 4). In the present study, the greater magnitude of improvement in the multimedia plus microteaching group suggests that teachers who underwent pedagogical skill reinforcement were more effective in translating oral health knowledge into daily classroom practices.

The sustained reduction in OHI-S scores over the six-month follow-up period indicates that behavioral changes were not merely short-term responses to the intervention. This finding is consistent with prior research emphasizing that continuous reinforcement by teachers plays a crucial role in maintaining positive oral hygiene behaviors among students, particularly in resource-limited school settings where access to dental professionals is limited (2, 10).

### Teachers' knowledge, attitudes, and practices (KAP)

The marked improvement in teachers' knowledge and instructional practices observed in this study supports existing literature that positions teachers as key agents of change in health promotion initiatives. Teacher-based interventions have been shown to be more effective when educators are adequately trained and confident in delivering health-related content (5, 6). While multimedia-based education has been widely recognized for enhancing knowledge acquisition through engaging visual and auditory content (8, 9), multimedia alone may be insufficient to ensure consistent and effective classroom implementation.

Microteaching appears to bridge this gap by enabling teachers to rehearse instructional delivery, receive structured feedback, and refine communication strategies. The significantly greater improvements in instructional behavior scores among teachers who participated in microteaching align with previous findings demonstrating that practicebased pedagogical training enhances teaching confidence and classroom performance (7, 12). This suggests that pedagogical competence, rather than knowledge alone, is critical for effective oral health promotion in school environments.

### Qualitative findings and mechanisms of sustained behavior change

The qualitative findings provide deeper insight into the mechanisms underlying the quantitative improvements observed. Teachers reported that microteaching facilitated the translation of theoretical knowledge into practical teaching strategies, enhanced confidence in responding to students' questions, and promoted consistency in oral health messaging. These findings corroborate educational theories emphasizing experiential learning and reflective practice as key drivers of sustained behavior change (12).

Moreover, the emergence of teachers' transformed roles—from information deliverers to active oral health facilitators—may explain the sustained behavioral improvements observed among students. Teachers' daily presence in schools allowed for continuous reinforcement of oral hygiene behaviors, such as proper toothbrushing techniques and dietary awareness. This mechanism aligns with behavior change models that emphasize repetition, social reinforcement, and role modeling as essential components for maintaining health-related behaviors over time (2, 11).

### Implications for scalability and sustainability

The integration of multimedia training with microteaching offers a practical and scalable model for oral health promotion in low-resource educational settings. Unlike dentist-led interventions that require repeated external inputs, this teacher-centered approach empowers schools to sustain oral health education independently. The findings support global recommendations advocating for the integration of oral health promotion into existing school curricula and teacher training frameworks (2).

### Study limitations

Several limitations should be considered when interpreting these findings. First, the study was conducted in a single district, which may limit generalizability to other regions. Second, teachers’ knowledge, attitudes, and practices were assessed using self-reported questionnaires, which may be subject to social desirability bias. Third, although the six-month follow-up period allowed for the assessment of short- to medium-term outcomes, longer follow-up durations are needed to evaluate the long-term sustainability of behavior change and oral health outcomes.

## Conclusion

In conclusion, integrating multimedia training with microteaching significantly improved teachers' instructional competence and students' oral health outcomes, as reflected by improvements in DMFT, OHI-S, and oral health–related knowledge, attitude, and behavior scores. This school-based intervention aligns with global recommendations from the World Health Organization emphasizing preventive oral health promotion in educational settings. Future research should explore longer follow-up periods, broader geographic implementation, and cost-effectiveness analyses to support large-scale adoption of this model.

## Data Availability

The original contributions presented in the study are included in the article/Supplementary Material, further inquiries can be directed to the corresponding author.
